# Deficiency of the lipid synthesis enzyme, DGAT1, extends longevity in mice

**DOI:** 10.18632/aging.100424

**Published:** 2012-01-29

**Authors:** Ryan S. Streeper, Carrie A. Grueter, Nathan Salomonis, Sylvaine Cases, Malin C. Levin, Suneil K. Koliwad, Ping Zhou, Matthew D. Hirschey, Eric Verdin, Robert V. Farese

**Affiliations:** ^1^ Gladstone Institute of Cardiovascular Disease, San Francisco, California, USA; ^2^ Gladstone Institute of Virology and Immunology, San Francisco, California, USA; ^3^ Cardiovascular Research Institute, San Francisco, California, USA; ^4^ Department of Medicine, University of California, San Francisco, California, USA; ^5^ Departments of Biochemistry and Biophysics, University of California, San Francisco, California, USA

**Keywords:** DGAT1, adipose tissue, longevity, triglycerides, calorie restriction

## Abstract

Calorie restriction results in leanness, which is linked to metabolic conditions that favor longevity. We show here that deficiency of the triglyceride synthesis enzyme acyl CoA:diacylglycerol acyltransferase 1 (DGAT1), which promotes leanness, also extends longevity without limiting food intake. Female DGAT1-deficient mice were protected from age-related increases in body fat, tissue triglycerides, and inflammation in white adipose tissue. This protection was accompanied by increased mean and maximal life spans of ~25% and ~10%, respectively. Middle-aged *Dgat1*^−/−^ mice exhibited several features associated with longevity, including decreased levels of circulating insulin growth factor 1 (IGF1) and reduced fecundity. Thus, deletion of DGAT1 in mice provides a model of leanness and extended lifespan that is independent of calorie restriction.

## INTRODUCTION

The amount of fat mass of an organism is emerging as key determinant in longevity. Too little or too much fat is associated with early mortality in rodents and humans, whereas leanness, intermediate with respect to these two extremes is associated with relative longevity, possibly reflecting an optimal amount of fat. The most effective intervention to promote leanness and increase lifespan is calorie restriction (CR) [1]. CR, with adequate nutrient intake, extends the lifespan of yeast, invertebrates (worm and fly) and mice [2-4], and is associated with favorable changes in energy metabolism [5]. However, CR requires markedly restricting food intake, which stimulates appetite and induces hunger, making CR difficult to maintain.

An alternative strategy for leanness is to limit the accumulation of body fat by activating energy expenditure. Although many interventions promote energy expenditure, we focus here on inhibiting acyl CoA:diacylglycerol acyltransferase 1 (DGAT1), which catalyzes the synthesis of triglycerides (TG) [6, 7]. We showed previously that male DGAT1-deficient (*Dgat1*^−/−^) mice have reduced adiposity associated with increased energy expenditure and normal-to-increased food intake [8, 9]. These mice also exhibit delayed intestinal fat absorption [10] and favorable metabolic changes, including enhanced insulin and leptin sensitivity [11, 12], resistance to diet-induced obesity, tissue steatosis, and glucose intolerance [8, 13]. Given these beneficial metabolic phenotypes, we hypothesized that DGAT1 deficiency protects against the metabolic consequences of aging and extends longevity. In this study, we examined lifespan and age-related changes in metabolism in female wild-type (WT) and *Dgat1*^−/−^ mice.

## RESULTS AND DISCUSSION

### *Dgat1*^−/−^ mice are protected against age-related metabolic changes

**Figure 1:**
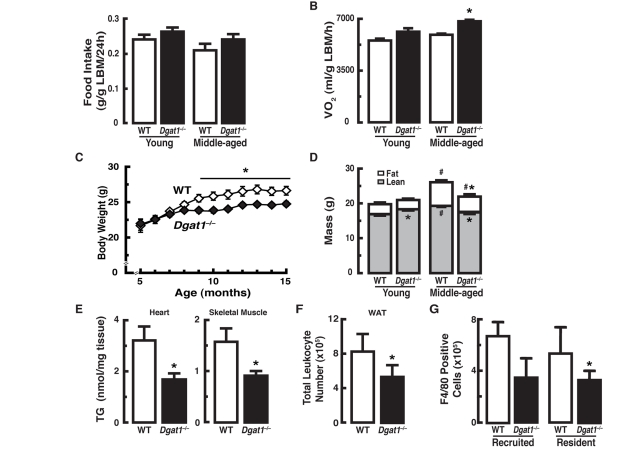
Female *Dgat1*^−/−^ mice have increased energy expenditure and are protected from changes in body composition associated with age (**A**) Normal food intake and (**B**) increased oxygen consumption in middle-aged *Dgat1*^−/−^ mice (n = 11-12/genotype). (**C**) Reduced body weight and (**D**) fat mass in *Dgat1*^−/−^ mice. Body weight was measured monthly in mice from the aging cohort (n = 30/genotype). Total body mass is expressed as fat and lean mass composition for young and middle-aged groups (n = 11-12/genotype). “Young” and “Middle-aged” refer to ages 3-4 months (mo) and 12-13 mo, respectively (*p < 0.05 *vs.* WT. ^#^*p* <0.05 vs. same genotype, different age). (**E**) Reduced levels of triglycerides in heart and gastrocnemius from middle-aged (15 mo) female mice [*p < 0.05 *vs.* wild-type (WT); n = 8-13]. The inguinal fat pads from *Dgat1*^−/−^ mice have fewer F4/80-positive (**F**) total leukocytes and (**G**) macrophages. (*p < 0.05 *vs.* WT; n = 5). F4/80-positive cells were separated into two populations, dim and bright, predicted to be the recruited and the resident macrophages, respectively [35]. Values are reported ± SEM.

We compared energy balance in young and middle-aged female WT and *Dgat1*^−/−^ mice fed a chow diet (fat content 10%). Food intake [g/g lean body mass (LBM)/24h] was similar in young (3-4 month-old) and middle-aged (12-15 month-old) groups of WT and *Dgat1*^−/−^ mice, though it trended higher in *Dgat1*^−/−^ mice (Figure 1A). Oxygen consumption (ml/g LBM/h; average 48 h), a measure of energy expenditure, was similar in young mice of either genotype, but was higher in middle-aged *Dgat1*^−/−^ mice (Figure 1B). This increase was present during both the light and dark cycles (data not shown). Although young WT and *Dgat1*^−/−^ mice had similar body weights (Figure 1C), the greater energy expenditure in *Dgat1*^−/−^ mice was associated with ~10% less body weight by 9 months of age that is sustained through middle age (Figure 1C).

Although bone mineral density (BMD), bone mineral content (BMC), and fat mass were similar in young mice of either genotype (Supplemental Figures 1A and B, and Figure 1D), lean mass was higher in young *Dgat1*^−/−^ mice (Figure 1D). In middle-aged WT mice, BMD, BMC (Supplemental Figures 1A and B) and lean and fat masses were increased compared to young WT mice (Figure 1D). In contrast, middle-aged *Dgat1*^−/−^ mice showed no increases in BMD, BMC (Supplemental Figures 1A and B) or lean mass and had smaller increases in fat mass (Figure 1D). In agreement with the lower fat mass and bone density, serum leptin levels were lower in middle-aged *Dgat1*^−/−^ mice than in WT controls (Supplemental Figure 1C).

The reduced fat mass of *Dgat1*^−/−^ mice was not confined to white adipose tissue (WAT), as middle-aged *Dgat1*^−/−^ mice also exhibited a ~40-50% lower levels of triglyceride (TG) in heart and skeletal muscle (Figure 1E). Liver TG content also trended lower in middle-aged *Dgat1*^−/−^ mice (~20%, *p* = 0.059, data not shown). TG accumulation in tissues can be associated with tissue inflammation, which has been identified as a signature of aging [14-16]. Indeed, despite the relatively low baseline of inflammation in mice fed a chow diet, the reduced adiposity seen in *Dgat1*^−/−^ mice was associated with fewer total leukocytes in inguinal fat (Figure 1F). This decrease was due, at least in part, to a reduction in the number of recruited and resident macrophages (F4/80-positive cells; Figure 1G). Of note, the weights of inguinal fat pads were essentially undistinguishable between the groups, consistent with our previous findings in non-pregnant *Dgat1*^−/−^ females [17]. Together, these data show that increased energy expenditure and reduced adiposity in middle-aged *Dgat1*^−/−^ mice is associated with less inflammation in the WAT.

### Female *Dgat1*^−/−^ mice have an extended lifespan

**Figure 2:**
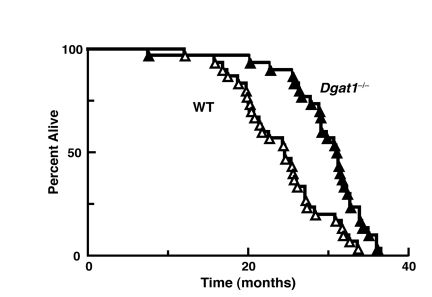
Extended longevity in female *Dgat1*^−/−^ mice Survival curves for female wild-type and *Dgat1*^−/−^ mice (n=30 per genotype). Further analysis of the data is summarized in Table 1.

We next determined if the leanness and reduction in age-related metabolic consequences in *Dgat1*^−/−^ miceaffected their longevity. Remarkably, the average lifespan in female *Dgat1*^−/−^ mice was ~5-6 months (~25%) longer than that for WT mice (Figure 2 and Table 1). The maximal and minimal lifespan (calculated as the mean of the oldest or youngest 10% of mice within a genotype) of *Dgat1*^−/−^ mice were also extended by ~3 and 6 months, respectively (Table 1; *p* < 0.0001).

**Table 1:** Comparative survival characteristics of female wild-type and *Dgat1*^−/−^ mice Oldest (youngest) 10% are the mean life span of the longest (or shortest) living 10% of animals within a genotype. Values are reported ±SEM, where appropriate (* *p*< 0.0001 *vs.* WT; n=30/genotype).

	Lifespan (days)				
	Mean	Median	Min-Max	Oldest 10%	Youngest 10%
**WT**	749 ± 32	746	480 - 1030	1014 ± 15	505 ± 54
***Dgat1*^−/−^**	920 ± 33*	942*	602 - 1114	1103 ± 7*	685 ± 62*
**% Increase**	23	26		9	11

**Figure 3:**
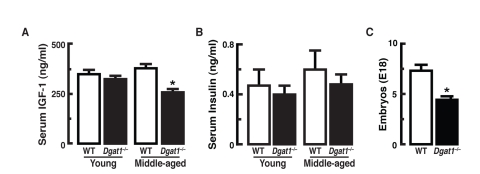
Longevity-related parameters in female *Dgat1*^−/−^ mice (**A**) Reduced serum IGF-1 levels in middle-aged *Dgat1*^−/−^ mice (*p*< 0.05; n= 9-10). (**B**) Similar serum insulin levels observed in young and middle-aged WT and *Dgat1*^−/−^ mice (n= 8-13). (**C**) Reduced fecundity in female *Dgat1*^−/−^ mice (*p<0.005; n= 11 litters/genotype). “Young” and “Middle-aged” refer to ages 3–4 mo and 14–16 mo, respectively. Values are reported ± SEM.

The extended longevity of female *Dgat1*^−/−^ mice was accompanied by factors that correlate with lifespan extension. Diminished insulin-like growth factor-1 (IGF-1)/insulin signaling can promote longevity in worms, flies and mice [5, 18-21]. Serum IGF-1 levels were similar in young WT and *Dgat1*^−/−^ mice, but were ~30% lower in middle-aged *Dgat1*^−/−^ mice than in WT controls (Figure 3A). Although fasting levels of blood glucose trended higher in young *Dgat1*^−/−^ mice and were significantly higher with age than in WT controls (data not shown), serum insulin levels (Figure 3B) and glucose tolerance (Supplemental Figure 2A) were similar in both groups of middle-aged *Dgat1*^−/−^ mice. This likely suggests that there were not large differences in insulin sensitivity. These findings in middle-aged, female mice contrast with those of young, male *Dgat1*^−/−^ mice [11], which are more insulin-sensitive, suggestive of a possible sexual dimorphism for this aspect of the DGAT1 deficiency phenotype.

Reduced fecundity also correlates with lifespan extension [22, 23]. We observed that *Dgat1*^−/−^ females had an average litter size of 3.8 pups compared with 7.4 pups for WT (Figure 3C). This observation is consistent with previous studies demonstrating an inverse relationship between reproduction and longevity [24].

### DGAT1 deficiency mimics some but not all aspects of calorie restriction

The finding that DGAT1 deficiency, like CR, extends longevity in mice prompted us to perform a more detailed phenotypic comparison of DGAT1-deficient mice with those subjected to CR. Beyond lifespan extension, many aspects of *Dgat1*^−/−^ mice (e.g., reduced adiposity, non-adipose tissue TG, tissue inflammation, bone density, fecundity, and decreased serum leptin and IGF1 levels) are similarly seen in CR [1, 5, 15, 19, 23, 25]. However, there were also notable physiological differences. First, *Dgat1*^−/−^ mice ate as much or more than WT mice (Figure 1A). In addition, hepatic mitochondrial biogenesis and function are increased during CR [26], possibly to reduce oxidative stress, but we found that mitochondrial DNA and citrate synthase activity were similar in the livers of middle-aged WT and *Dgat1*^−/−^ mice (Supplemental Figure 2B and C).

To further investigate the changes in response to CR and DGAT1 deficiency, we performed whole-genome microarray and pathway analyses on liver samples of WT mice fed a calorie restricted diet and *Dgat1*^−/−^ mice fed *ad libitum*. Middle-aged female WT mice were subjected to short-term CR (WTCR), in which intake was restricted by 25% for 2 weeks and 50% for the following 2 weeks (achieving ~3 g of weight loss every 2 weeks), while female middle-aged *Dgat1*^−/−^ mice were fed *ad libitum* (*Dgat1*^−/−^AL). To identify genes whose mRNA levels were significantly altered, both groups were compared with WT controls fed *ad libitum* (WTAL).

**Figure 4:**
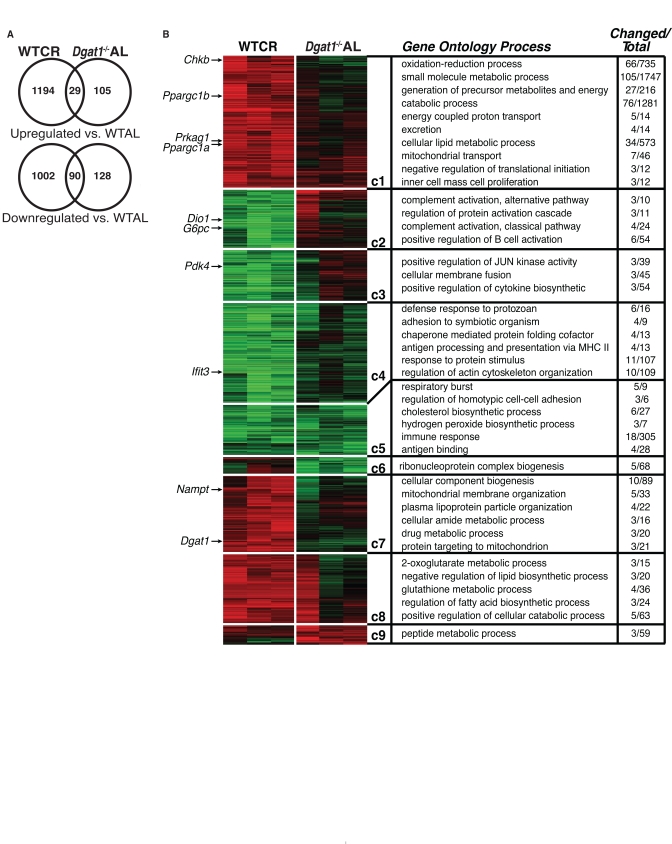
Comparative gene expression and pathway analysis of calorie restricted and *Dgat1*-deficient female mice (**A**) Differentially expressed genes (fold>1.3, non-adjusted t-test *p*<0.05) in the livers of WT calorie restricted (WTCR) *vs.* WT ad libitum mice (WTAL) compared to *Dgat1*^−/−^ ad libitum (*Dgat1*^−/−^ AL) *vs.* WTAL. (**B**) Gene-expression profiles (vertical axis) and biological replicates (horizontal axis) are shown in the context of the HOPACH cluster map. Ordered root HOPACH clusters (C1-9) represent significantly up-regulated (red) or down-regulated (green) genes relative to WTAL (WTAL data excluded). Over-represented cluster Gene Ontology and pathway terms (GO-Elite) are shown for the top-ranking distinct processes based on a permuted *p*-value. *Dgat1* and genes previously associated with CR are indicated by an arrow next to the location of the corresponding gene probe set in the cluster map.

A subset of genes with altered mRNA levels was validated by quantitative RT-PCR (Supplemental Figure 3). Among these validated genes, we found that DGAT1 expression was up-regulated ~twofold in response to CR. Overall, nearly seven times as many genes were altered with CR than in *Dgat1*^−/−^AL (Figure 4 and Supplemental Table 1), suggesting that CR and DGAT1 deficiency alter physiology and promote lifespan extension in different ways. Still, we discovered a relatively small subset of about 100 commonly up- or down-regulated genes (Figure 4, Supplemental Table 1). These genes are of potential interest since they might point to common pathways that promote murine longevity. Notably, both CR and DGAT1 deficiency caused significant down-regulation of immune/inflammation response and cholesterol biosynthesis pathways (Figure 4, clusters C4 and C5). Although several genes involved in inflammation and immune responses were similarly regulated (*Emr1, Ccl5, C1qa and Ccl3*; Supplemental Table 1), the most highly down-regulated transcripts were *Fabp5, Igj* and *Cxcl13* (Supplemental Table 1). These findings are consistent with the decreased levels of markers of inflammation in WAT of middle-aged *Dgat1*^−/−^ mice (Figure 1F and G). Both WTCR and *Dgat1^−/−^AL* mice showed down-regulation of genes involved in the cholesterol biosynthesis pathway (*Fdft1*, *Fdps*, *Hmgcs1, Mvd*, and *Sqle*; Supplemental Figure 4), a finding that was also observed in the livers of long-lived Snell dwarf (*Pit1^dw/dwJ^*) and ribosomal protein S6 kinase 1 knockout mice *(S6K1*^−/−^) mice [27, 28]. Furthermore, circulating levels of total cholesterol were ~35% lower in middle-aged female *Dgat1*^−/−^ mice than WT controls (39 ± 3 vs. 61 ± 5 mg/dL in WT; *p* < 0.01). Of note, DGAT1 deficiency in apolipoprotein E-deficient mice decreases cholesterol absorption, blood cholesterol, and foam cell formation, resulting in protection from atherosclerosis [29]. Thus, DGAT1 deficiency, like CR, may promote favorable changes in lipid metabolism that promote extended lifespan.

### Summary and Implications

Our findings show that deletion of the TG synthesis enzyme, DGAT1, promotes leanness and extends lifespan in female mice and therefore suggests a link between murine lipid metabolism and longevity. These results are consistent with a study linking lipid metabolism and longevity in *Caenorhabditis elegans*, where activation of lipid hydrolysis resulted in decreased fat mass and extended lifespan [30]. We presume that the effects of DGAT1 deficiency are the result of reduced TG and related lipid metabolites in tissues. However, notably, DGAT1 has several biochemical activities [6], and we therefore cannot exclude the possibility that changes in other DGAT1-associated pathways contribute to these effects.

We propose that DGAT1 deficiency, by increasing energy expenditure and maintaining leanness, activates a metabolic program favorable for extending lifespan without restricting calories. Although the details of this process remain unclear, both DGAT1 deficiency and CR alter parameters related to cholesterol metabolism and inflammation, two processes linked to atherosclerosis and cardiovascular disease. The phenotype of *Dgat1*^−/−^ mice is similar to that of mice lacking insulin receptors in WAT, which are also lean, have increased energy expenditure and food consumption and exhibit extended longevity [31, 32]. The physiological alterations in both of these models may limit the accumulation of body fat and protect from the adverse metabolic consequences of excessive fat storage. Our findings suggest that inhibition of DGAT1, or other strategies to promote leanness, may have the potential to retard age-related metabolic disease and prolong lifespan in humans.

## METHODS

### Mice

*Dgat1*^−/−^ mice (C57BL/6J background) were generated as described [8]. For weight and survival curve studies, mice were housed in a conventional room. For all other studies, mice were housed in a pathogen-free barrier-type facility (both with a 12-h light/12-h dark cycle). Female mice were used for all studies and were fed a standard chow diet (5053 PicoLab Diet; Purina, St. Louis, MO). For the short-term calorie restriction study, we compared three groups of 15-16-month-old mice: WT-calorie restricted (WTCR), WT-ad libitum (WTAL) and *Dgat1*^−/−^ -ad *libitum* (*Dgat1*^−/−^AL). Mice were individually housed and given free access to water. After a baseline assessment of average daily calorie consumption, WTCR mice were switched from ~106 kcal/d to 80 kcal/d of CR diet for 2 weeks, followed by 53 kcal/d for 2 weeks. For fecundity studies, timed matings were performed with young 8-12-week-old virgin female WT and *Dgat1*^−/−^ mice and proven male breeders. Because *Dgat1*^−/−^ mice do not lactate, embryos were harvested at day 18 of pregnancy, and the number of offspring was recorded.

### Body composition

Mice were fasted for 4 h and anesthetized, and their body compositions were analyzed by dual energy X-ray absorptiometry (DEXA) with a PixiMus2 scanner (GE Healthcare Lunar, Madison, WI).

### Energy balance

Food intake (powdered standard chow) and oxygen consumption (*V*O_2_) were measured simultaneously using the Comprehensive Lab Animal Monitoring System (Columbus Instruments, Columbus, OH). Animals were acclimated to the cages on day one, and data were recorded on days two and three. Both food intake and *V*O_2_ were normalized to lean body mass, as measured by DEXA scanning on the day before calorimetry studies.

### Lipid analyses

Tissue TG were analyzed as described [33]. Briefly, lipids were extracted from heart, skeletal muscle and liver homogenates in CHCl_3_:MeOH [2:1(v/v)]and separated by thin-layer chromatography with hexane:ethyl ether:acetic acid [80:20:1(v/v)]on silica gel G-60 TLC plates.

### Tissue inflammation

Total leukocytes and macrophages were assessed from pooled inguinal fat pads of 2 virgin female mice (4 pads per sample) fed a chow diet. Fat pads were minced with a razor blade, digested for 2 h at 37°C with 1 mg/ml type III collagenase (Worthington Biochemicals) and 100 U/ml DNase in DMEM/F12 containing 3% fatty acid-free BSA, and washed in BSA-containing medium. The cellular pellet was collected, incubated for 10 min at RT in ACK lysis buffer and washed again in staining buffer. Total leukocytes were counted at this point. To determine the proportion of macrophages in the isolated leukocyte population, cells were plated in 96-well V-bottom culture plates and incubated with rat anti-mouse CD16/CD32 (1:500 in staining buffer; BD Pharmingen, San Diego, CA) for 30 min at 4°C to block Fc receptors. Cells were then washed once with staining buffer, incubated for 30 min at 4°C with PE-conjugated F4/80 (BD), washed twice with staining buffer and fixed in 1% paraformaldehyde in PBS at 4°C until analysis with a FACS Calibur flow cytometer (BD Biosciences). Data were acquired with CellQuest software (BD Biosciences) and analyzed with FlowJo software (TreeStar, Mountain View, CA). F4/80 positive cells were separated into two populations, dim and bright. The F4/80 bright population is predicted to be the resident macrophages and the F4/80 dim population the recruited macrophages [34].

### Serum analytes

Serum insulin (Linco, St. Louis, MO) and IGF1 (Diagnostics Systems Laboratories, Inc. Webster, TX) and leptin (R&D Systems, Minneapolis, MN) levels were measure by ELISA according to the manufacturer's instructions.

### Mitochondrial content

Liver tissues were collected from 14 month old mice and mitochondrial content was measured. Briefly, the liver was homogenized in Tris buffer [50 mM Tris-HCl (pH 7.5) containing 10 mM EDTA, 250 mM sucrose, pH 7.5]. The homogenate was centrifuged at 1000*g* for 5 min at 4°C, thereby pelleting genomic DNA. The supernatant was then centrifuged at 10,000*g* for 30 min at 4°C to pellet the mitochondria. This pellet was resuspended in lysis buffer [10 mM Tris-HCl (pH 8.0), 20 mM EDTA, 0.5% Triton X-100)] and placed on ice for 20 min. Protein K (14 mg/ml) and RNase (10 mg/ml) were added to each sample and incubated overnight at 50°C. Mitochondrial DNA was then extracted using an equal volume of phenol/chloroform and 1/5 volume of 5 mM NaCl, and precipitated in an equal volume of isopropanol at −20°C overnight. After centrifugation at 12,000*g* at room temperature, the resulting pellet of mtDNA was washed with 70% ethanol and then dried. The pellet was resuspended in 10 mM Tris-HCl buffer, pH 8.0, containing 1 mM EDTA and 20 mg/ml RNase. Genomic DNA content did not differ among groups.

### Biochemical assays

Citrate synthase activity was measured by monitoring the conversion of acetyl-CoA and oxaloacetate to citrate, using the CoA thiol reaction with 5,5'-dithio-2-nitrobenzoate (DNTB) as described [35].

### Gene expression analyses

mRNA levels were quantified as described [33]. Oligonucleotide primers were designed using Primer Bank (Supplemental Table 2) [36]. For microarray studies, three mice per group at 15-16 months of age were sacrificed, and their livers were flash frozen. Total RNA was isolated from approximately 100 mg of homogenized liver and prepared for hybridization to Mouse Affymetrix Gene 1.0 ST arrays, according to the manufacturer's protocol. The gene expression data were deposited in the Gene Expression Omnibus database (GSE26267). Expression values were obtained using RMA [37] and associated non-adjusted t-test p-values with the limma R package [38]. Expression clustering was performed using HOPACH [39]. Pathway analysis and visualization were performed with the software GenMAPP-CS (http://www.genmapp.org/beta/genmappcs).

### Glucose tolerance

After an overnight fast, mice were injected intraperitoneally with glucose (2 g/kg body weight), and blood glucose was measured at 0, 15, 30, 60, and 120 min with a One-Touch UltraSmart glucose monitoring system (Lifescan, Milpitas, CA).

### Statistical analyses

Data are presented as mean ± SEM. Survival curves were analyzed using the Kaplan-Meier method. Means were compared with a Mann-Whitney rank-sum test or by analysis of variance followed by a Student Newman-Keuls multiple comparisons test. Weight curves were compared with a repeated measures ANOVA test followed by a Newman-Keuls test.

## SUPPLEMENTAL DATA

Supplemental Figure 1 Changes in bone mineral density, bone mineral content and leptin levels in middle-aged *Dgat1*^−/−^ mice (**A**) Bone mineral density and (**B**) bone mineral content are lower in middle-aged *Dgat1*^−/−^ versus WT mice. (**C**) Serum leptin levels are lower in middle-aged female mice [*p < 0.05 *vs.* wild-type (WT); n = 8–13]. “Young” and “Middle-aged” refer to ages 3–4 mo and 14–16 mo, respectively.

Supplemental Figure 2 Similar glucose tolerance and hepatic mitochondrial content in middle-aged WT and *Dgat1*^−/−^ mice (**A**) Blood glucose levels of middle-aged mice before and after an intraperitoneal injection of glucose (1 mg/g body weight). Mice were fed regular chow and fasted 5–6 hours before the test. (**B**) Mitochondrial (mt) DNA content and (**C**) citrate synthase (CS) activity, a marker of mitochondrial activity, from the livers of middle-aged mice (n=4 and 10, respectively).

Supplemental Figure 3 Validation of microarray anlaysis with qRT-PCR Differentially expressed genes in the livers of WTCR and *Dgat1*^−/−^ AL *vs.* WT ad libitum mice. Values are mean ± SEM of biological triplicates.

Supplemental Figure 4 Analysis of cholesterol biosynthesis pathway Cholesterol biosynthesis pathway-highlighted genes (WikiPathways: WP103, revision 41337) that are significantly down-regulated in WTCR (left) or KOAL (right) relative to WTAL based on pathway analysis from the program GenMAPP-CS.

## References

[R1] Weindruch R, Walford L (1998). The Retardation of Aging and 4Diseases by Dietary Restriction.

[R2] Piper M, Bartke A (2008). Diet and aging. Cell Metab.

[R3] Kennedy BK, Steffen K, Kaeberlein M (2007). Ruminations on dietary restriction and aging. Cell Mol Life Sci.

[R4] Koubova J, Guarente L (2003). How does calorie restriction work?. Genes Dev.

[R5] Anderson RM, Weindruch R (2010). Metabolic reprogramming, caloric restriction and aging. Trends Endocrinol Metab.

[R6] Yen C-LE, Monetti M, Burri BJ, Farese RV (2005). The triacylglycerol synthesis enzyme DGAT1 also catalyzes the synthesis of diacylglycerols, waxes, and retinyl esters. J Lipid Res.

[R7] Yen C, Stone S, Koliwad S, Harris C, Farese RV (2008). Thematic review series: glycerolipids. DGAT enzymes and triacylglycerol biosynthesis. J Lipid Res.

[R8] Smith SJ, Cases S, Jensen DR, Chen HC, Sande E, Tow B, Sanan DA, Raber J, Eckel RH, Farese RV (2000). Obesity resistance and multiple mechanisms of triglyceride synthesis in mice lacking DGAT. Nat Genet.

[R9] Chen HC, Ladha Z, Smith SJ, Farese RV (2003). Analysis of energy expenditure at different ambient temperatures in mice lacking DGAT1. Am J Physiol Endocrinol Metab.

[R10] Buhman KK, Smith SJ, Stone SJ, Repa JJ, Wong JS, Knapp FF, Burri BJ, Hamilton RL, Abumrad NA, Farese RV (2002). DGAT1 is not essential for intestinal triacylglycerol absorption or chylomicron synthesis. J Biol Chem.

[R11] CChen HC, Smith SJ, Ladha Z, Jensen DR, Ferreira LD, Pulawa LK, McGuire JG, Pitas RE, Eckel RH, Farese RV (2002). Increased insulin and leptin sensitivity in mice lacking acyl CoA:diacylglycerol acyltransferase 1. J Clin Invest.

[R12] Chen HC, Ladha Z, Farese RV (2002). Deficiency of acyl coenzyme a:diacylglycerol acyltransferase 1 increases leptin sensitivity in murine obesity models. Endocrinology.

[R13] Chen HC, Jensen DR, Myers HM, Eckel RH, Farese RV (2003). Obesity resistance and enhanced glucose metabolism in mice transplanted with white adipose tissue lacking acyl CoA:diacylglycerol acyltransferase 1. J Clin Invest.

[R14] Slawik M, Vidal-Puig AJ (2006). Lipotoxicity, overnutrition and energy metabolism in aging. Ageing Res Rev.

[R15] Unger RH (2005). Longevity, lipotoxicity and leptin: the adipocyte defense against feasting and famine. Biochimie.

[R16] Finch CE, Crimmins EM (2004). Inflammatory exposure and historical changes in human life-spans. Science.

[R17] Cases S, Zhou P, Shillingford JM, Wiseman BS, Fish JD, Angle CS, Hennighausen L, Werb Z, Farese RV (2004). Development of the mammary gland requires DGAT1 expression in stromal and epithelial tissues. Development.

[R18] Katic M, Kahn C (2005). The role of insulin and IGF-1 signaling in longevity. Cell Mol Life Sci.

[R19] Barger JL, Walford RL, Weindruch R (2003). The retardation of aging by caloric restriction: its significance in the transgenic era. Exp Gerontol.

[R20] Kenyon C (2005). The plasticity of aging: insights from long-lived mutants. Cell. Mol Life Sci.

[R21] Longo VD, Finch CE (2003). Evolutionary medicine: From dwarf model systems to healthy centenarians?. Science.

[R22] Mukhopadhyay A, Tissenbaum HA (2007). Reproduction and longevity: secrets revealed by C. elegans. Trends Cell Biol.

[R23] Chen Y (2010). Longevity and lifespan control in mammals: lessons from the mouse. Ageing Research Reviews.

[R24] Kirkwood T (1977). Evolution of ageing. Nature.

[R25] Bluher M (2008). Fat Tissue and Long Life. The European Journal of Obesity.

[R26] López-Lluch G, Hunt N, Jones B, Zhu M, Jamieson H, Hilmer S, Cascajo MV, Allard J, Ingram DK, Navas P, de Cabo R (2006). Calorie restriction induces mitochondrial biogenesis and bioenergetic efficiency. Proc Natl Acad Sci.

[R27] Selman C, Tullet JM, Wieser D, Irvine E, Lingard SJ, Choudhury AI, Claret M, Al-Qassab H, Carmignac D, Ramadani F, Woods A, Robinson IC, Schuster E, Batterham RL, Kozma SC, Thomas G, Carling D, Okkenhaug K, Thornton JM, Partridge L, Gems D, Withers DJ Ribosomal protein S6 kinase 1 signaling regulates mammalian life span. Science.

[R28] Boylston WH, Gerstner A, DeFord JH, Madsen M, Flurkey K, Harrison DE, Papaconstantinou J (2004). Altered cholesterologenic and lipogenic transcriptional profile in livers of aging Snell dwarf (Pit1dw/dwJ) mice. Aging Cell.

[R29] Chandak PG, Obrowsky S, Radovic B, Doddapattar P, Aflaki E, Kratzer A, Doshi LS, Povoden S, Ahammer H, Hoefler G, Levak-Frank S, Kratky D (2011). Lack of acyl-CoA:diacylglycerol acyltransferase 1 reduces intestinal cholesterol absorption and attenuates atherosclerosis in apolipoprotein E knockout mice. Biochim Biophys Acta.

[R30] Wang M, O'Rourke E, Ruvkun G (2008). Fat metabolism links germline stem cells and longevity in C. elegans. Science.

[R31] Blüher M, Kahn BB, Kahn CR (2003). Extended longevity in mice lacking the insulin receptor in adipose tissue. Science.

[R32] Katic M, Kennedy AR, Leykin I, Norris A, McGettrick A, Gesta S, Russell SJ, Bluher M, Maratos-Flier E, Kahn CR (2007). Mitochondrial gene expression and increased oxidative metabolism: role in increased lifespan of fat-specific insulin receptor knock-out mice. Aging Cell.

[R33] Levin MC, Levin MC, Monetti M, Watt MJ, Sajan MP, Stevens RD, Bain JR, Newgard CB, Farese RV, Farese RV (2007). Increased lipid accumulation and insulin resistance in transgenic mice expressing DGAT2 in glycolytic (type II) muscle. Am J Physiol Endocrinol Metab.

[R34] Peters W, Cyster JG, Mack M, Schlöndorff D, Wolf AJ, Ernst JD, Charo IF (2004). CCR2-dependent trafficking of F4/80dim macrophages and CD11cdim/intermediate dendritic cells is crucial for T cell recruitment to lungs infected with Mycobacterium tuberculosis. J Immunol.

[R35] Srere P (1969). Citrate Synthase. Methods Enzymol.

[R36] Wang X, Seed B (2003). A PCR primer bank for quantitative gene expression analysis. Nucleic Acids Res.

[R37] Irizarry R, Bolstad BM, Collin F, Cope LM, Hobbs B, Speed TP (2003). Summaries of Affymetrix GeneChip probe level data. Nucleic Acids Res.

[R38] Duboit S, Gentleman R, Quackenbush J (2003). Open source software for the analysis of microarray data. Bio Techniques.

[R39] Pollard K, van der Laan MJ (2002). A method to identify significant clusters in gene expression data. Proceedings of SCI 2001.

