# Supplementary figures and images for: Deficiency of the lipid synthesis enzyme, DGAT1, extends longevity in mice
Source: Aging (Albany NY). 2012 Jan 29;4(1):13–27. doi: 10.18632/aging.100424 (PMC3292902; doi:10.18632/aging.100424)

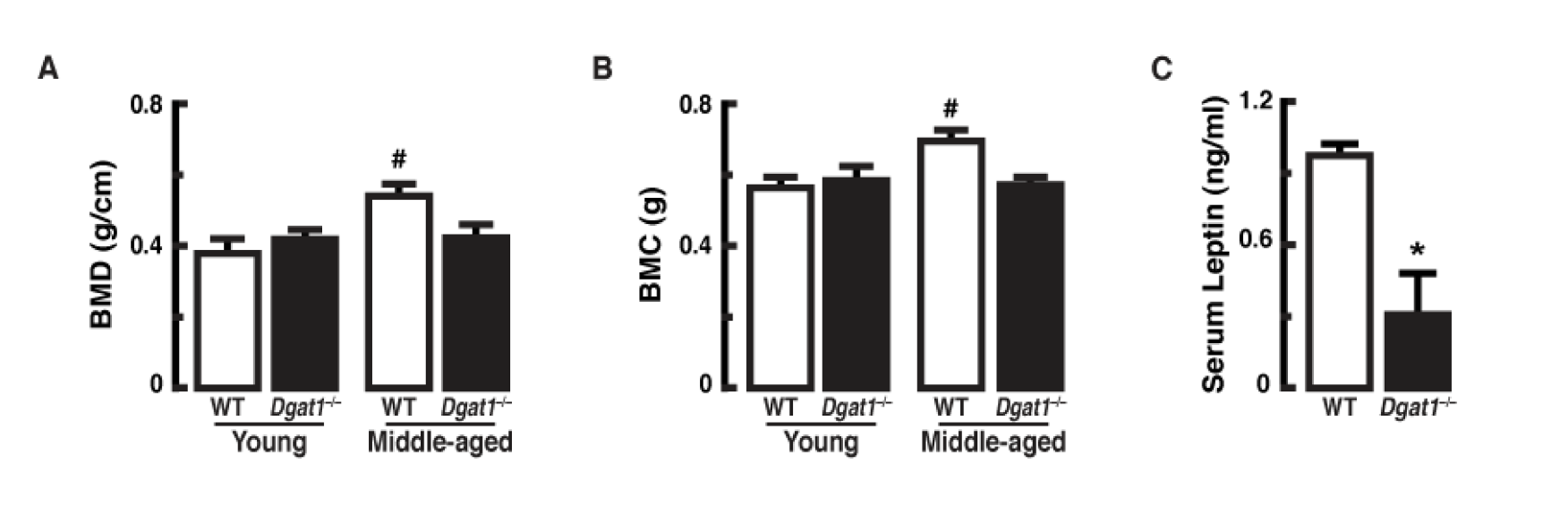

Supplement: Supplemental Figure 1 — (A) Bone mineral density and (B) bone mineral content are lower in middle-aged Dgat1−/− versus WT mice. (C) Serum leptin levels are lower in middle-aged female mice [*p < 0.05 vs. wild-type (WT); n = 8–13]. “Young” and “Middle-aged” refer to ages 3–4 mo and 14–16 mo, respectively. [file aging-04-013-s001.tif]

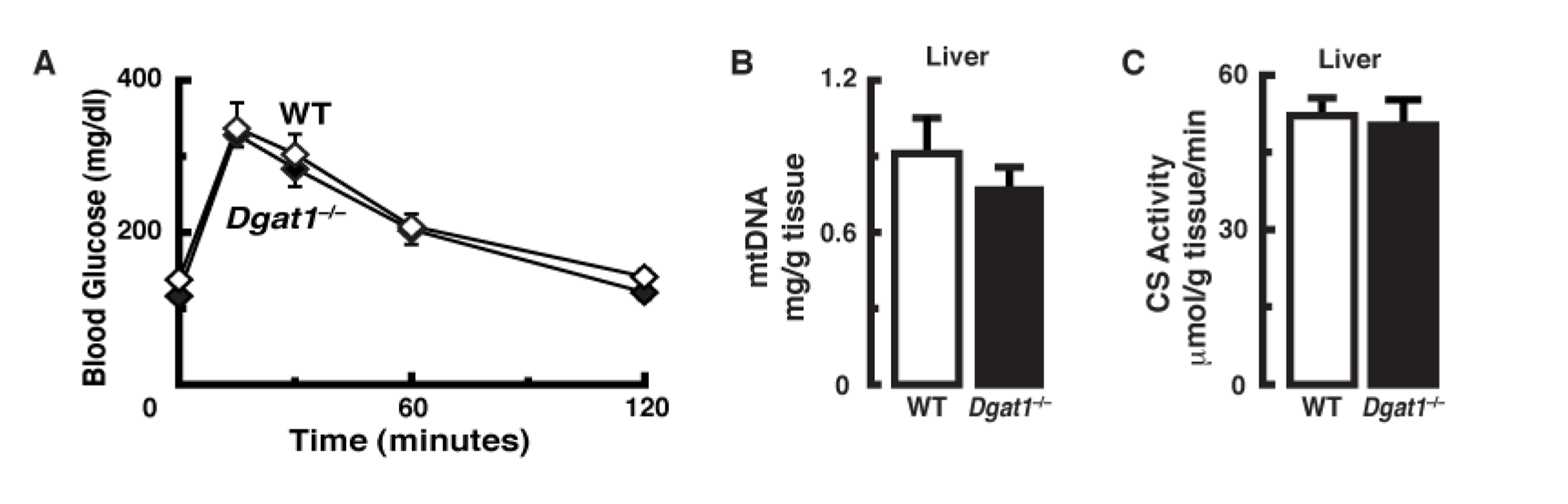

Supplement: Supplemental Figure 2 — (A) Blood glucose levels of middle-aged mice before and after an intraperitoneal injection of glucose (1 mg/g body weight). Mice were fed regular chow and fasted 5–6 hours before the test. (B) Mitochondrial (mt) DNA content and (C) citrate synthase (CS) activity, a marker of mitochondrial activity, from the livers of middle-aged mice (n=4 and 10, respectively). [file aging-04-013-s002.tif]

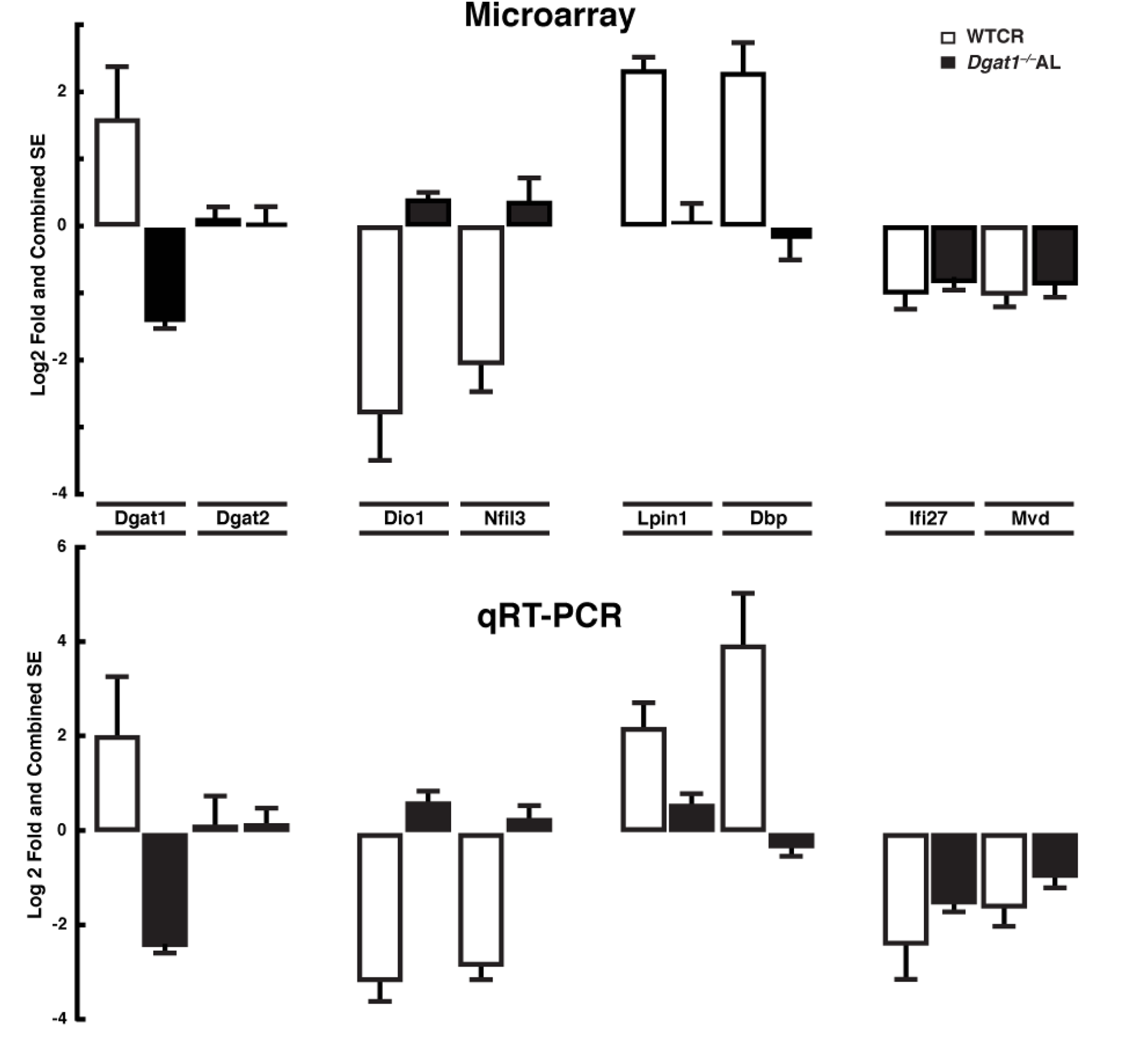

Supplement: Supplemental Figure 3 — Differentially expressed genes in the livers of WTCR and Dgat1−/− AL vs. WT ad libitum mice. Values are mean ± SEM of biological triplicates. [file aging-04-013-s003.tif]

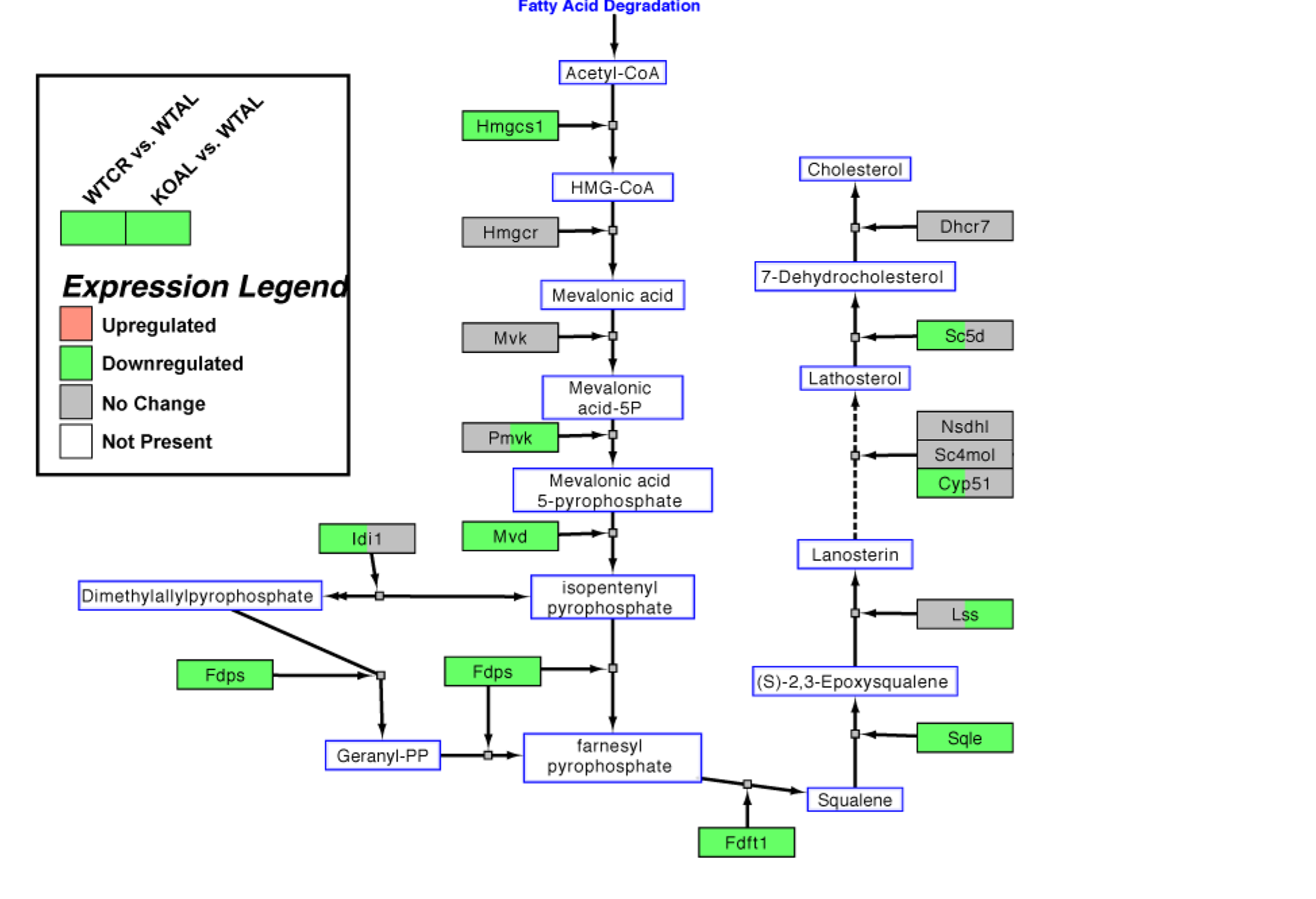

Supplement: Supplemental Figure 4 — Cholesterol biosynthesis pathway-highlighted genes (WikiPathways: WP103, revision 41337) that are significantly down-regulated in WTCR (left) or KOAL (right) relative to WTAL based on pathway analysis from the program GenMAPP-CS. [file aging-04-013-s004.tif]
