# Supplementary material for: Deficiency of the lipid synthesis enzyme, DGAT1, extends longevity in mice
Source: Aging (Albany NY). 2012 Jan 29;4(1):13–27. doi: 10.18632/aging.100424 (PMC3292902; doi:10.18632/aging.100424)
Supplement: Supplementary file 5 [file aging-04-013-s001.doc]

**Supplemental Data**

**Deficiency of the lipid synthesis enzyme, DGAT1, extends longevity in mice**

Ryan S. Streeper^1,3*^, Carrie A. Grueter^1*^, Nathan Salomonis^1^, Sylvaine Cases^1^, Malin C. Levin^1^, Suneil K. Koliwad^1,3,4^, Ping Zhou^1^, Matthew D. Hirschey^2^, Eric Verdin^2,4^, and Robert V. Farese, Jr.^1,3,4,5^,

1Gladstone Institute of Cardiovascular Disease, San Francisco, California, USA

2Gladstone Institute of Virology and Immunology, San Francisco, California, USA

3Cardiovascular Research Institute, San Francisco, California, USA

4Department of Medicine, University of California, San Francisco, California, USA

5Departments of Biochemistry and Biophysics, University of California, San Francisco, California, USA

*These authors contributed equally.

This supplement contains:

Supplemental Figures 1-4

Supplemental Table 1

Supplemental Figure Legends

**
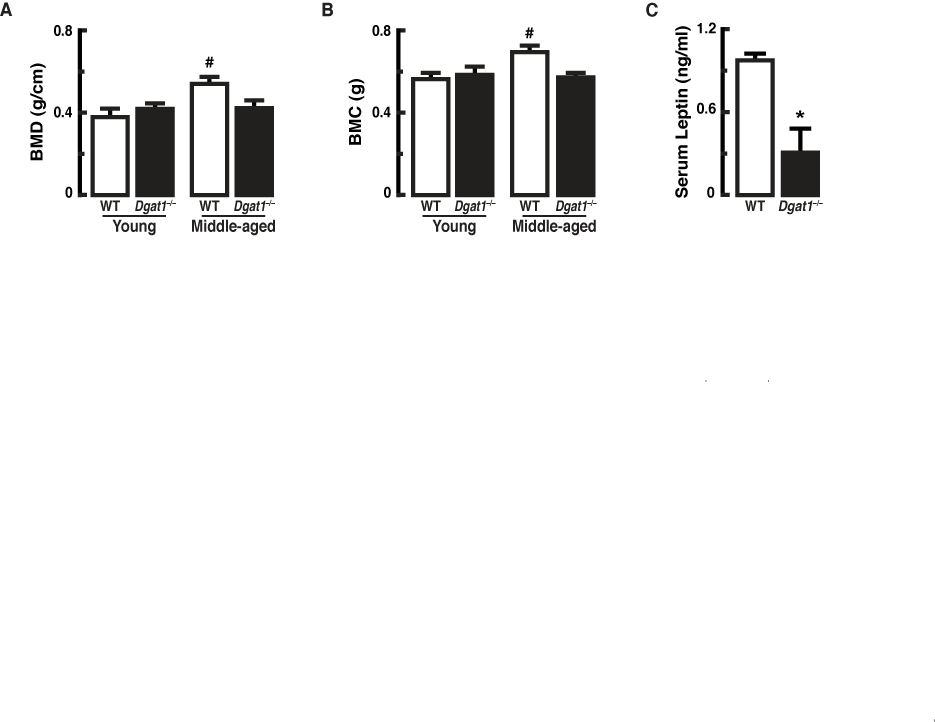
**

**Supplemental Figure 1. Changes in bone mineral density, bone mineral content and leptin levels in middle-aged *Dgat1****^–/–^* **mice.** (**A**) Bone mineral density and (**B**) bone mineral content are lower in middle-aged *Dgat1*^−/−^ versus WT mice. (**C**) Serum leptin levels are lower in middle-aged female mice [**p* < 0.05 *vs.* wild-type (WT); n = 8–13]. “Young” and “Middle-aged” refer to ages 3–4 mo and 14–16 mo, respectively.

**
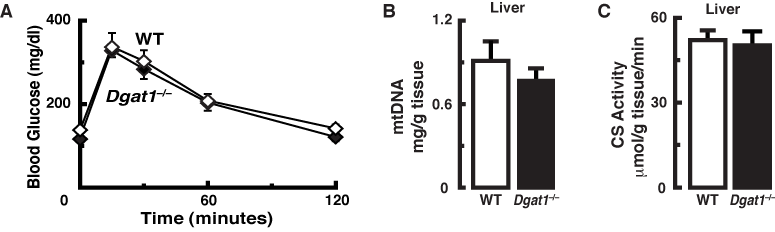
**

**Supplemental Figure 2. Similar glucose tolerance and hepatic mitochondrial content in middle-aged WT and *Dgat1****^–/–^* **mice.** (**A**) Blood glucose levels of middle-aged mice before and after an intraperitoneal injection of glucose (1 mg/g body weight). Mice were fed regular chow and fasted 5–6 hours before the test. (**B**) Mitochondrial (mt) DNA content and (**C**) citrate synthase (CS) activity, a marker of mitochondrial activity, from the livers of middle-aged mice (n=4 and 10, respectively).

**
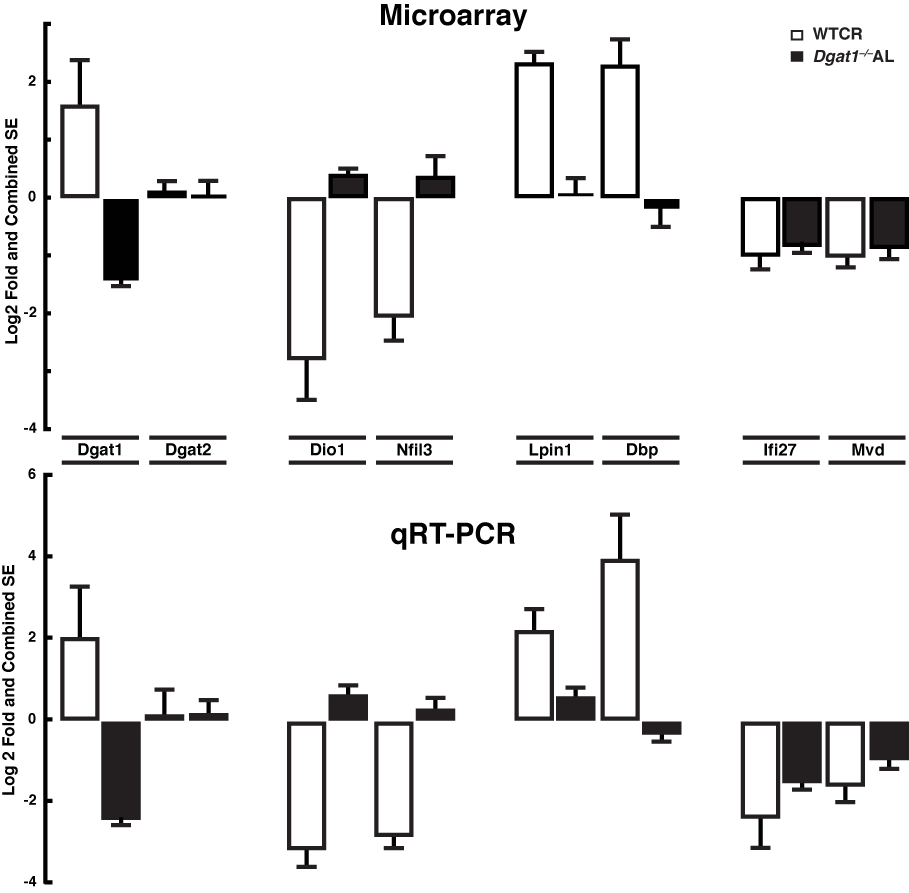
**

**Supplemental Figure 3. Validation of microarray anlaysis with qRT-PCR.** Differentially expressed genes in the livers of WTCR and *Dgat1^–/–^* AL *vs.* WT ad libitum mice. Values are mean ± SEM of biological triplicates.

**
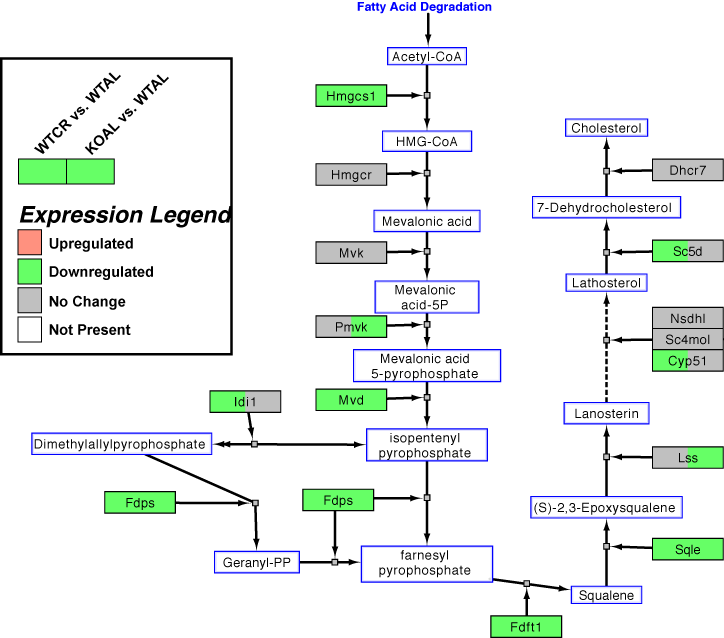
**

**Supplemental Figure 4. Analysis of cholesterol biosynthesis pathway.** Cholesterol biosynthesis pathway-highlighted genes (WikiPathways: WP103, revision 41337) that are significantly down-regulated in WTCR (left) or KOAL (right) relative to WTAL based on pathway analysis from the program GenMAPP-CS.

**Supplemental Table 1.**

**A. Genes commonly up-regulated in liver of short-term calorie restricted wild-type (WTCR) and ad libitum *Dgat1^–/–^*** (KOAL) middle-aged (15-16 mo) female mice.

| **ProbeSetID** | **GeneSymbol** | **fold.WTCR-WTAL** | **fold.KOAL-WTAL** | **rawP.WTCR-WTAL** | **rawP.KOAL-WTAL** |
| --- | --- | --- | --- | --- | --- |
| 10349431 | Acmsd | 1.51 | 2.15 | 2.4E-02 | 8.0E-04 |
| 10488608 | Trib3 | 1.83 | 1.78 | 4.8E-03 | 6.2E-03 |
| 10496077 | Agxt2l1 | 1.95 | 1.78 | 3.8E-04 | 1.0E-03 |
| 10593937 | Mpi | 1.52 | 1.47 | 9.5E-03 | 1.4E-02 |
| 10589099 | Ihpk2 | 1.72 | 1.44 | 8.8E-04 | 9.2E-03 |
| 10519607 | 4930420K17Rik | 1.42 | 1.43 | 2.2E-03 | 2.1E-03 |
| 10503023 | Cth | 1.31 | 1.43 | 9.8E-03 | 2.1E-03 |
| 10441359 | --- | 1.49 | 1.42 | 6.4E-03 | 1.3E-02 |
| 10368947 | Aim1 | 2.04 | 1.39 | 5.0E-05 | 7.6E-03 |
| 10571891 | Aadat | 1.69 | 1.39 | 3.3E-03 | 3.3E-02 |
| 10399430 | Ddx1 | 1.37 | 1.38 | 1.2E-02 | 1.2E-02 |
| 10464754 | Rhod | 1.96 | 1.37 | 7.6E-04 | 4.3E-02 |
| 10378568 | --- | 1.58 | 1.37 | 1.0E-03 | 9.4E-03 |
| 10434289 | --- | 1.55 | 1.36 | 1.2E-04 | 1.4E-03 |
| 10574641 | D230025D16Rik | 1.52 | 1.36 | 2.4E-03 | 1.4E-02 |
| 10585823 | LOC665268 | 1.36 | 1.35 | 3.0E-03 | 3.5E-03 |
| 10593671 | Dmxl2 | 1.75 | 1.34 | 7.0E-04 | 2.4E-02 |
| 10515187 | Cyp4a14 | 2.60 | 1.34 | 2.9E-05 | 4.0E-02 |
| 10409021 | Tpmt | 1.34 | 1.33 | 3.1E-03 | 3.4E-03 |
| 10357878 | Adora1 | 1.60 | 1.33 | 2.0E-03 | 2.9E-02 |
| 10354233 | Tgfbrap1 | 1.32 | 1.33 | 4.5E-02 | 4.1E-02 |
| 10581340 | Ranbp10 | 1.34 | 1.32 | 2.3E-02 | 3.0E-02 |
| 10398195 | Ccnk | 1.32 | 1.32 | 1.3E-02 | 1.3E-02 |
| 10593668 | Dmxl2 | 1.54 | 1.31 | 4.6E-03 | 4.0E-02 |
| 10410124 | Ctsl | 1.48 | 1.31 | 2.5E-03 | 1.7E-02 |
| 10475247 | Tmem62 | 1.35 | 1.31 | 2.7E-02 | 3.8E-02 |
| 10436200 | EG667802 | 1.44 | 1.31 | 1.3E-02 | 4.9E-02 |
| 10485622 | Qser1 | 1.34 | 1.31 | 6.3E-03 | 9.9E-03 |
| 10420216 | 2310014G06Rik | 1.60 | 1.31 | 1.3E-04 | 5.0E-03 |

**B. Genes commonly down-regulated in liver of short-term calorie restricted wild-type (WTCR) and ad libitum Dgat1-/- (KOAL) middle-aged (15-16 mo) female mice.**

| **ProbeSetID** | **GeneSymbol** | **fold.WTCR-WTAL** | **fold.KOAL-WTAL** | **rawP.WTCR-WTAL** | **rawP.KOAL-WTAL** |
| --- | --- | --- | --- | --- | --- |
| 10531126 | Igj | -5.01 | -2.86 | 1.1E-03 | 1.3E-02 |
| 10523359 | Cxcl13 | -2.80 | -2.52 | 2.6E-02 | 4.1E-02 |
| 10585699 | ENSMUST00000098689 | -3.58 | -2.26 | 1.9E-04 | 3.7E-03 |
| 10509163 | Id3 | -2.29 | -2.19 | 8.3E-04 | 1.2E-03 |
| 10490838 | Fabp5 | -3.41 | -2.14 | 4.2E-04 | 8.0E-03 |
| 10425161 | Lgals1 | -1.62 | -2.00 | 4.5E-02 | 8.9E-03 |
| 10361246 | G0s2 | -2.51 | -1.95 | 1.6E-04 | 1.4E-03 |
| 10425822 | Pnpla3 | -1.99 | -1.94 | 3.2E-02 | 3.8E-02 |
| 10603860 | Cfp | -1.50 | -1.87 | 4.3E-02 | 5.5E-03 |
| 10587082 | Onecut1 | -1.69 | -1.82 | 1.5E-03 | 6.1E-04 |
| 10446282 | Emr1 | -2.19 | -1.80 | 2.7E-03 | 1.3E-02 |
| 10365830 | --- | -1.53 | -1.77 | 2.2E-02 | 5.0E-03 |
| 10582310 | Mvd | -1.95 | -1.77 | 5.3E-04 | 1.5E-03 |
| 10402347 | Ifi27 | -1.94 | -1.73 | 2.9E-04 | 1.1E-03 |
| 10407281 | Esm1 | -1.53 | -1.72 | 1.7E-03 | 3.3E-04 |
| 10571321 | Ppp1r3b | -1.43 | -1.69 | 2.3E-02 | 3.1E-03 |
| 10385504 | EG432555 | -1.91 | -1.69 | 2.5E-04 | 1.0E-03 |
| 10438405 | Igl-V1 /// Igl-J2 /// Igl-V2 /// LOC433053 | -1.88 | -1.66 | 9.3E-04 | 3.5E-03 |
| 10501063 | Cd53 | -1.59 | -1.64 | 1.0E-02 | 7.5E-03 |
| 10517165 | Cd52 | -2.16 | -1.63 | 1.2E-05 | 3.4E-04 |
| 10455015 | --- | -3.90 | -1.63 | 1.1E-04 | 4.2E-02 |
| 10404606 | Ly86 | -1.85 | -1.61 | 2.9E-03 | 1.2E-02 |
| 10461614 | Ms4a6c | -2.76 | -1.61 | 2.8E-05 | 5.0E-03 |
| 10385518 | Tgtp /// OTTMUSG00000005523 | -3.25 | -1.61 | 6.6E-05 | 1.9E-02 |
| 10458890 | --- | -1.41 | -1.59 | 4.1E-03 | 6.2E-04 |
| 10545187 | EG628498 /// Gm1502 /// Igkv4-74 /// Gm1524 /// LOC100047316 | -1.93 | -1.59 | 6.9E-04 | 5.8E-03 |
| 10538903 | ENSMUSG00000076577 /// Igk-V28 /// Igkv6-25 /// Igk-V21-4 /// Igk /// Gm1499 /// LOC676193 | -2.10 | -1.59 | 4.7E-04 | 8.5E-03 |
| 10466200 | Ms4a7 | -1.69 | -1.58 | 7.9E-03 | 1.6E-02 |
| 10499483 | Fdps | -1.39 | -1.58 | 3.0E-02 | 6.1E-03 |
| 10403034 | Igh /// LOC676399 /// LOC100046275 | -1.74 | -1.57 | 1.4E-02 | 3.5E-02 |
| 10385533 | Tgtp /// OTTMUSG00000005523 | -2.81 | -1.56 | 3.2E-04 | 3.5E-02 |
| 10603551 | Cybb | -1.82 | -1.56 | 2.9E-03 | 1.4E-02 |
| 10444258 | Psmb8 | -2.08 | -1.56 | 1.1E-04 | 3.1E-03 |
| 10403063 | Igh /// LOC100046275 /// LOC676399 | -1.57 | -1.54 | 1.1E-02 | 1.4E-02 |
| 10368240 | Tcf21 | -1.40 | -1.53 | 1.3E-02 | 3.7E-03 |
| 10517517 | C1qa | -1.45 | -1.53 | 1.9E-02 | 9.6E-03 |
| 10545237 | --- | -1.37 | -1.53 | 6.3E-03 | 1.1E-03 |
| 10403743 | Inhba | -1.42 | -1.52 | 2.2E-03 | 7.1E-04 |
| 10531724 | Plac8 | -2.20 | -1.52 | 1.6E-03 | 4.0E-02 |
| 10444291 | H2-Ab1 /// Rmcs5 /// Rmcs2 | -3.02 | -1.52 | 8.5E-06 | 6.9E-03 |
| 10450154 | H2-Aa | -3.57 | -1.52 | 2.1E-05 | 2.4E-02 |
| 10545198 | EG667683 /// Igk /// Gm1499 /// Gm1524 | -1.79 | -1.51 | 2.3E-03 | 1.5E-02 |
| 10545184 | EG628498 /// Igkv4-74 /// Gm189 /// Gm1524 /// LOC100047316 | -2.02 | -1.51 | 2.7E-04 | 7.8E-03 |
| 10503359 | C430048L16Rik | -1.67 | -1.50 | 4.4E-04 | 2.2E-03 |
| 10412466 | Hmgcs1 | -2.41 | -1.49 | 1.6E-04 | 1.8E-02 |
| 10496592 | Gbp2 | -2.00 | -1.49 | 2.1E-03 | 3.5E-02 |
| 10545196 | Igk-C /// Gm1524 /// Igk /// Gm1499 /// Gm1418 /// Gm189 /// Igkv4-74 | -1.57 | -1.49 | 1.5E-04 | 3.7E-04 |
| 10379630 | Slfn2 | -1.89 | -1.49 | 7.0E-05 | 1.8E-03 |
| 10556018 | --- | -1.49 | -1.48 | 1.8E-02 | 1.9E-02 |
| 10576774 | Clec4g | -2.00 | -1.48 | 1.7E-05 | 1.1E-03 |
| 10389231 | Ccl3 | -1.38 | -1.47 | 1.5E-02 | 6.0E-03 |
| 10539433 | Mobkl1b | -1.59 | -1.47 | 1.3E-02 | 3.1E-02 |
| 10559467 | Pira11 /// Pira6 /// Pira4 /// Pira3 /// Pira7 /// OTTMUSG00000022068 /// Pira2 /// Lilrb3 | -1.66 | -1.45 | 3.4E-04 | 2.5E-03 |
| 10582303 | Cyba | -1.46 | -1.45 | 1.0E-02 | 1.0E-02 |
| 10389207 | Ccl5 | -1.89 | -1.45 | 1.7E-03 | 2.9E-02 |
| 10466606 | Anxa1 | -1.35 | -1.45 | 3.3E-02 | 1.3E-02 |
| 10424349 | Sqle | -1.35 | -1.44 | 2.0E-02 | 8.3E-03 |
| 10568024 | Coro1a | -1.62 | -1.41 | 6.2E-04 | 4.7E-03 |
| 10401181 | Rdh11 | -1.69 | -1.41 | 2.0E-03 | 1.9E-02 |
| 10607865 | Tmsb4x | -2.23 | -1.41 | 2.4E-05 | 7.2E-03 |
| 10545731 | Clec4f | -1.69 | -1.41 | 5.6E-04 | 7.3E-03 |
| 10569646 | Ccnd1 | -4.08 | -1.41 | 2.0E-06 | 2.4E-02 |
| 10421648 | Slc25a30 | -2.16 | -1.40 | 1.8E-04 | 2.5E-02 |
| 10545202 | Gm1077 /// Gm1418 | -1.39 | -1.39 | 1.6E-02 | 1.6E-02 |
| 10555087 | --- | -1.37 | -1.39 | 1.3E-02 | 1.0E-02 |
| 10420730 | Fdft1 | -1.32 | -1.39 | 2.9E-02 | 1.3E-02 |
| 10360382 | Ifi204 /// Mnda /// Ifi205 | -2.45 | -1.38 | 1.1E-04 | 4.3E-02 |
| 10503995 | Dnaja1 | -1.81 | -1.38 | 1.8E-03 | 4.3E-02 |
| 10358224 | Ptprc | -2.43 | -1.37 | 5.8E-05 | 3.0E-02 |
| 10444298 | H2-Eb1 | -2.43 | -1.36 | 1.1E-04 | 4.7E-02 |
| 10539135 | Capg | -1.35 | -1.35 | 6.2E-03 | 5.9E-03 |
| 10367050 | Rdh18 | -1.31 | -1.35 | 3.3E-02 | 2.1E-02 |
| 10473125 | Itga4 /// Cerkl | -1.52 | -1.35 | 8.7E-04 | 6.5E-03 |
| 10480699 | Dpp7 | -1.65 | -1.35 | 2.0E-04 | 5.3E-03 |
| 10569102 | Irf7 | -1.56 | -1.35 | 6.6E-03 | 4.0E-02 |
| 10548892 | Arhgdib | -1.53 | -1.35 | 7.7E-04 | 6.8E-03 |
| 10356020 | Dock10 | -1.94 | -1.34 | 6.3E-07 | 3.2E-04 |
| 10531952 | Abcg3 | -1.87 | -1.34 | 5.4E-04 | 3.4E-02 |
| 10412207 | 2310016C16Rik | -1.44 | -1.34 | 7.4E-03 | 2.1E-02 |
| 10366640 | 1190005P17Rik | -1.35 | -1.34 | 8.2E-03 | 9.0E-03 |
| 10385513 | EG432555 /// 9930111J21Rik /// Psme2b-ps /// RP23-269N23.3 | -1.91 | -1.34 | 4.0E-05 | 8.0E-03 |
| 10367066 | Rdh20 | -3.46 | -1.32 | 1.7E-06 | 3.2E-02 |
| 10563441 | Emp3 | -1.41 | -1.32 | 4.5E-03 | 1.4E-02 |
| 10524621 | Oasl2 | -1.55 | -1.32 | 7.4E-04 | 1.1E-02 |
| 10603573 | Sytl5 | -1.91 | -1.32 | 1.8E-05 | 6.2E-03 |
| 10395612 | 6030408C04Rik | -1.35 | -1.31 | 1.4E-02 | 2.2E-02 |
| 10466210 | Ms4a6d | -1.37 | -1.31 | 2.8E-03 | 6.9E-03 |
| 10500335 | Fcgr1 | -1.43 | -1.31 | 9.0E-03 | 3.4E-02 |
| 10403079 | LOC435333 | -1.51 | -1.30 | 5.2E-04 | 7.9E-03 |
| 10530910 | Uba6 | -1.77 | -1.30 | 6.3E-04 | 4.1E-02 |

**Supplementary** **Table 2.** Primers used for quantitative RT-PCR analysis.

| **Gene** | **Sequence** |
| --- | --- |
| *Dgat1* | F: 5'- GACGGCTACTGGGATCTGA -3'  R: 5'- TCACCACACACCAATTCAGG -3' |
| *Dgat2* | F: 5'- CGCAGCGAAAACAAGAATAA -3'  R: 5'- GAAGATGTCTTGGAGGGCTG -3' |
| *Dio1* | F: 5'- ccctggtgttgaactttggc -3'  R: 5'- tgaggaaatcggctgtgga -3' |
| *Nfil3* | F: 5'- CTTTCTTTTCCCCCTCACG -3'  R: 5'- CATCCATCAATGGGTCCTTC -3' |
| *Lpin1* | F: 5'- TTTTTGCATACAAAGGCAGC -3'  R: 5'- TTCACCGTCACAAACACCTG -3' |
| *Dpb* | F: 5'- cgcgcaggcttgacatcta -3'  R: 5'- tgaggaaatcggctgtgga-3' |
| *Ifi27* | F: 5'- GTTGGGAACACTGTTTGGCT -3'  R: 5'- CAATGCCTGTCCCAGTGAA -3' |
| *Mvd* | F: 5'- CGGTCAACATCGCAGTTATC - 3'  R: 5'- TCGTTTTTAGCTGGTCCTGG -3' |
